# Intraoperative Angle Measurement of Anatomical Structures: A Systematic Review

**DOI:** 10.3390/s24051613

**Published:** 2024-03-01

**Authors:** João Cruz, Sérgio B. Gonçalves, Manuel Cassiano Neves, Hugo Plácido Silva, Miguel Tavares Silva

**Affiliations:** 1 IDMEC, Instituto Superior Técnico, Universidade de Lisboa, Av. Rovisco Pais 1, 1049-001 Lisboa, Portugal; joaopiresdacruz@tecnico.ulisboa.pt (J.C.); sergio.goncalves@tecnico.ulisboa.pt (S.B.G.); 2 Hospital CUF Descobertas, R. Mário Botas, 1998-018 Lisboa, Portugal; manuel.cassianoneves@cuf.pt; 3 IT—Instituto de Telecomunicações, Instituto Superior Técnico, Universidade de Lisboa, Av. Rovisco Pais 1, 1049-001 Lisboa, Portugal; hugo.placido.silva@tecnico.ulisboa.pt

**Keywords:** angle measurement, biomedical sensors, intraoperative, rotational deformities, angular deformities, surgery, osteotomy

## Abstract

Ensuring precise angle measurement during surgical correction of orientation-related deformities is crucial for optimal postoperative outcomes, yet there is a lack of an ideal commercial solution. Current measurement sensors and instrumentation have limitations that make their use context-specific, demanding a methodical evaluation of the field. A systematic review was carried out in March 2023. Studies reporting technologies and validation methods for intraoperative angular measurement of anatomical structures were analyzed. A total of 32 studies were included, 17 focused on image-based technologies (6 fluoroscopy, 4 camera-based tracking, and 7 CT-based), while 15 explored non-image-based technologies (6 manual instruments and 9 inertial sensor-based instruments). Image-based technologies offer better accuracy and 3D capabilities but pose challenges like additional equipment, increased radiation exposure, time, and cost. Non-image-based technologies are cost-effective but may be influenced by the surgeon’s perception and require careful calibration. Nevertheless, the choice of the proper technology should take into consideration the influence of the expected error in the surgery, surgery type, and radiation dose limit. This comprehensive review serves as a valuable guide for surgeons seeking precise angle measurements intraoperatively. It not only explores the performance and application of existing technologies but also aids in the future development of innovative solutions.

## 1. Introduction

Orientation-related deformities are a common occurrence among young children, especially on the lower extremities [1,2]. The limbs naturally go through changes during embryonic limb development and, after birth, during childhood. Therefore, it is to be expected that deformities may happen [1,2,3]. These can manifest as angular (deformity in the sagittal plane, coronal plane, or in a plane oblique to the coronal or sagittal planes), rotational or longitudinal (deformity in the transverse or horizontal plane), or often as a combination of both types [4].

Although most instances of congenital deformities are resolved naturally and do not persist past adolescence, they can still exist in adults [1,2,3]. Additionally, deformities are not exclusively a congenital condition and can be found also as a result of trauma or in a pathological context [3,5]. In the best case scenarios, the deformities naturally correct, and it is possible to live without knowing there ever was such a problem. However, there are cases where the deformities have been linked with pain and changes in biomechanical parameters, such as movement impairments, limb range of motion (RoM) deficiencies, and muscle incapabilities [3,5,6,7]. When the deformities lead to those conditions, a surgical correction is sometimes necessary [1,2]. Different procedures exist, but the main corrective procedure is the osteotomy.

An osteotomy is a surgical procedure that aims to realign or reshape a bone by cutting it, ensuring predictable new bone formation and healing to correct the deformity [8]. It is a procedure that can be applied to many different bones of the human body, namely, in the head [9,10,11], torso [12,13,14], upper limbs [15,16,17,18], and lower limbs [19,20,21,22]. Osteotomy surgical procedures vary in technique, with mainly two purposes: realigning the bone and bone lengthening or transport [8]. There are also several different bone fixation methods that maintain the cut bone in the correct healing position, ranging from internal ones, such as screws [23,24,25], pins [23,26], and plates [23,24], to external ones, such as the Ilizarov external fixator [23,27], the Taylor spatial frame [28], and the unilateral Hifixator [29]. Like most of the medical field, it is a procedure that has evolved with time, from being first reported by Hippocrates, circa 415 BC [8,30], until today. Major developments in preoperative planning, intraoperative guidance, and postoperative care have been achieved. With that in mind, there is still a lot in this field that needs to be investigated.

Preoperative planning of the osteotomy is an important part of the procedure, defining the correction needed and enabling the surgeon to simulate a deformity correction ahead of the surgical procedure [31,32,33]. Available preoperative planning systems include manual instruments [34] and imaging instruments such as radiographs [35,36], fluoroscopy [37], ultrasound [38], magnetic resonance imaging (MRI) [39], and computed tomography (CT) [36,40]. However, that planning, on its own, does not guarantee the ideal outcome, as several studies have reported differences between the preoperative target correction and the postoperative outcome [41,42]. This highlights the value that intraoperative angle adjustments can have in order to achieve the postoperative angle desired, assisting the surgeons in maintaining a correction angle in line with the preoperative target [43,44].

Currently, there are some limitations associated with intraoperative angle adjustments, as most systems imply an increase in complexity and invasiveness, prolonged intervention time, special equipment, exposure to radiation, increased cost, a learning curve, and may be unavailable in some hospitals [43,44,45]. Moreover, there is difficulty in using the same system for different types of osteotomies and different places, as these conditions may imply a higher degree of difficulty in obtaining angle measurements [20,21,22,44]. These limitations create a problem for a surgeon performing an osteotomy, as the intraoperative adjustments will frequently have to rely on the surgeon’s best estimate of the angles [46,47] since the drawbacks of the available systems render them unusable for most situations. Therefore, there is an unanswered need that requires further developments in the intraoperative measurement systems field, allowing for the real-time acquisition of segment orientation and their relative position.

When designing an intraoperative system capable of addressing these limitations and relaying to the surgeon the necessary information for optimal angle adjustments, it is of the utmost importance to be aware of the available tools for intraoperative angle measurements as part of the design process [48]. To that end, the present work aims to gather a thorough and structured account of the state-of-the-art intraoperative angle measurement techniques that are reported, comparing the techniques’ advantages and disadvantages. Aiming to expand the scope of applications and explore possible approaches to address this challenge, the present analysis will not be limited solely to solutions that have been used in osteotomy surgeries. Instead, it will encompass methodologies utilized intraoperatively to measure angles between various anatomical structures in diverse surgical contexts, including body segments, bones, and soft tissues.

This research is of notable value due to the absence of an optimal commercial solution addressing the challenge of intraoperative angle measurement. Consequently, a comprehensive analysis of the existing technologies, their target, advantages, and limitations is important. Furthermore, the gathering of the existing body of knowledge on this topic may have a dual purpose. On the one hand, it can assist surgeons in their selection of appropriate technology for their procedures. On the other hand, it may facilitate the validation of existing theoretical tools as well as the development of potentially innovative solutions. With that purpose, a systematic review following the Preferred Reporting Items for Systematic Reviews and Meta-Analyses (PRISMA) statement [49] was performed. Five electronic databases were searched, and studies deemed eligible were included. Additionally, the articles in the reference lists of those studies were also reviewed, and those that met the eligibility criteria were incorporated into this review.

The remainder of this article is organized as follows: Section 2 presents the search strategy employed, including databases, keywords and search phrases, and the number of obtained articles. It continues describing the screening and selection process, including the eligibility criteria defined that lead to the articles included in the review. Then, Section 3 showcases the relevant data extracted from the obtained articles, the identified technologies, and their respective performance. Section 4 offers a discussion of the technologies identified as well as a look into the limitations of the study and future work opportunities. Finally, in Section 5, conclusions of the work developed are presented.

## 2. Methods

### 2.1. Search Strategy

With the goal of identifying studies that evaluated intraoperative angle measurement techniques applied in a surgery context, a rigorous search on five electronic databases (PubMed, Scopus, Web of Science, Cochrane Library, and IEEE Xplore) was carried out on 30 March 2023, without any publication year restrictions.

The search strategy was established by first defining the following primary keywords: (i) surgery, (ii) angle, (iii) measurement, and (iv) intraoperative. Given that the results obtained from the combination of these keywords were too wide-ranging, for each of these four primary keywords, a group of keywords was created with commonly used words in place of the respective primary keyword, as shown in Table 1.

Initially, keywords inside each of the created groups were linked using the OR Boolean operator with the field tags [Title/Abstract], [Topic], [Article Title/Abstract/Keywords], [Title/Abstract/Keywords], and [All Metadata] on PubMed, Scopus, Web of Science, Cochrane Library, and IEEE Xplore, respectively. The groups were then linked using the AND Boolean operator. To prevent the appearance of articles that were not relevant to the research question of this work, an additional restriction was created to only obtain articles with the phrase “angle measurement” or any phrase obtained by combining each keyword from the angle group with the measurement group by utilizing quotation marks. In an effort to allow for different variations of the phrase, words from the measurement group were truncated by utilizing the asterisk (*). For example, combining the word “orientation”, from the angle group, with the truncated words from the measurement group resulted in the following ten phrases: “orientation measure*”; “orientation determin*”; “orientation calculate*”; “orientation comput*”; “orientation assess*”; “orientation evaluat*”; “orientation estimate*”; “orientation mensurat*”; “orientation quantif*”; and “orientation valuat*”.

This process created 50 different phrases that were combined using the OR Boolean operator, thus creating a new group in the place of the angle and measurement groups, to be combined with the surgery and intraoperative groups using the AND Boolean operator. A detailed overview of the search strategy, including the results for every keyword, phrase, and combination of groups using Boolean operators is presented in Table S1 provided in Supplementary Materials. For the IEEE Xplore database, since it only allows for eight wildcard terms, only eight keywords could be truncated. Hence, an adjustment had to be made, and only the top eight phrases that returned the most results were kept truncated.

The database search returned a total of 291 articles, with the following distribution: 42 from PubMed, 163 from Scopus, and 86 from Web of Science. However, no results were obtained from the Cochrane Library or IEEE Xplore.

### 2.2. Study Screening and Selection

Following the database search, all records were extracted simultaneously to an Excel 365 (Microsoft^®^, Redmond, WA, USA) file, as well as to the Covidence online software (Covidence, Melbourne, Australia). The latter is self-described as a web-based collaboration software platform that streamlines the production of systematic and other literature reviews [50]. Using the software, duplicates were identified and manually confirmed, resulting in the removal of 118 duplicate studies. From the remaining 173 studies, titles, abstracts, and keywords were screened, producing 58 articles that were retrieved and their full text examined and subjected to the eligibility criteria.

This process was conducted by two reviewers: one with a biomedical engineering background and the other a practicing orthopedist with over 30 years of experience. Disagreements were resolved by a third reviewer.

Prior to the search procedure, inclusion and exclusion criteria were defined to establish an article’s eligibility for this review. Studies were included if they met all the following criteria: (i) the study reported an angle measurement instrumentation, technique, or technology; (ii) said technology is used intraoperatively and (iii) provides real-time information to the surgeon; and (iv) the study does not meet any exclusion criteria. Importantly, a study that reported a navigation or guidance system that is capable of angle measurement, but does not report on the angle measurement technique or technology, does not meet inclusion criteria (i) and therefore is not included.

On the other hand, a study was excluded if it met any of the following criteria: (i) does not report an angle measurement technique or technology or (ii) reports the use of an angle measurement technique or technology but does not provide data on its use; (iii) does not report intraoperative use of the technique or technology; (iv) mentioned intraoperative use but only reported preoperative and/or postoperative data or (v) provides insufficient data on its intraoperative use; (vi) is a systematic review or meta-analyses; and (vii) is not written in English. Additionally, during the full-text reading, it was necessary to add supplementary exclusion criteria: (viii) if the article reports exclusively on intraoperative angle measurement of tools and not limbs; and (ix) if the full text could not be retrieved. For example, the study by Zhou et al. [51] reports on intraoperative angle measurement and meets inclusion criteria (i–iii); however, it focuses on guidewire position analysis and therefore is excluded according to criterion (viii). Carli et al. [52] studied inconsistencies between navigation data and radiographs in total knee arthroplasty and reported intraoperative measurements of the navigation system, but it does not focus on these measures and they are simply used as a comparison and so, understandably, does not provide sufficient data on the navigation system’s intraoperative use and is therefore excluded according to criterion (v).

**Figure 1:**
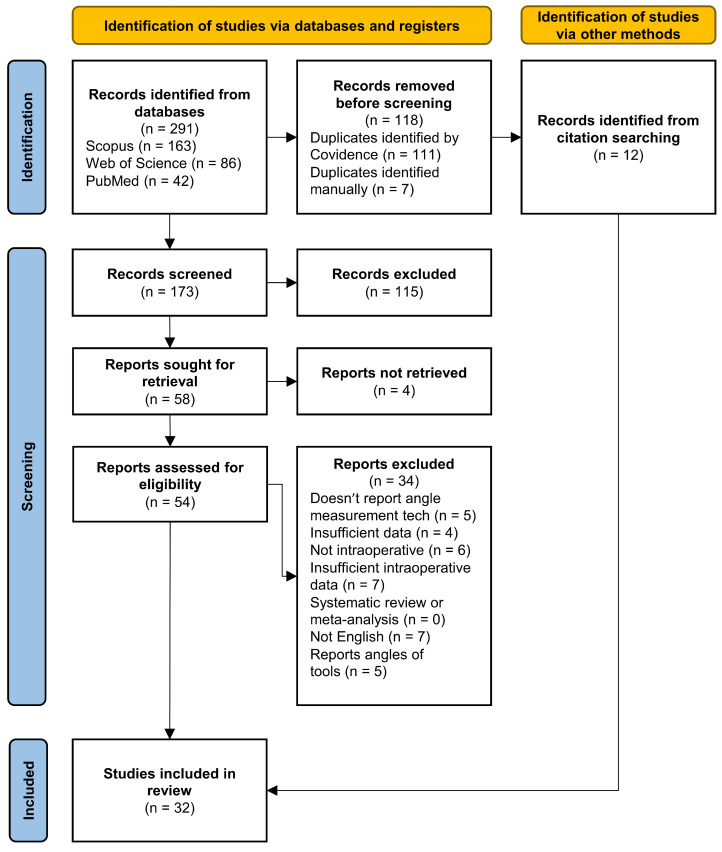
Flowchart of the search conducted, detailing the screening process, created with Covidence [50].

A flowchart of the search methodology described in this section is available in Figure 1. Of the 58 articles, a total of 20 met the eligibility criteria and were included in the review. The references of those articles were carefully examined, and 12 additional relevant studies were identified that met the criteria and were therefore also added, resulting in a total of 32 studies to be analyzed.

**Figure 2:**
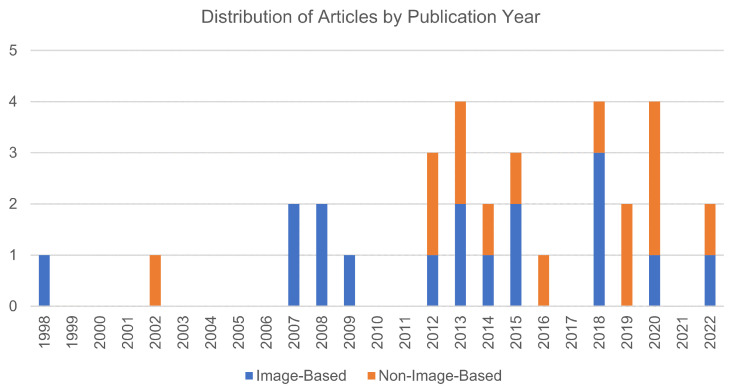
Analysis of publication year distribution for the included studies, categorizing them into the two groups: image-based studies (depicted by the blue bars) and non-image-based studies (represented by the orange bars).

The next sections will present the findings of the review, grouping the techniques in image-based or non-image-based. Some studies mentioned more than one angle measurement technique for comparison. Those will be placed in the group where the main focus of the article is while also referencing the comparisons. The studies will be organized in chronological order, and the pertinent data will be presented, specifically encompassing details such as the employed technology, surgical target and/or type, and the principal validation outcomes. In total, 17 articles refer to image-based techniques, and 15 refer to non-image-based techniques. The distribution of the articles by publication year, specifying their group, is illustrated in Figure 2.

## 3. Results

### 3.1. Image-Based Technologies

For the purpose of this review, a technique was considered image-based if the angle measurement was obtained from an image or the angle was calculated based on data obtained from an image. Some articles mentioned more than one technology as a comparison. In these cases, the articles are grouped considering the technology that is the main focus of study.

Additionally, certain articles mentioned multiple angle measurement technologies. To maintain consistency with the previously described grouping criterion, they are categorized alongside the imaging technology that provides the data from which the angles are derived.

**Figure 3:**
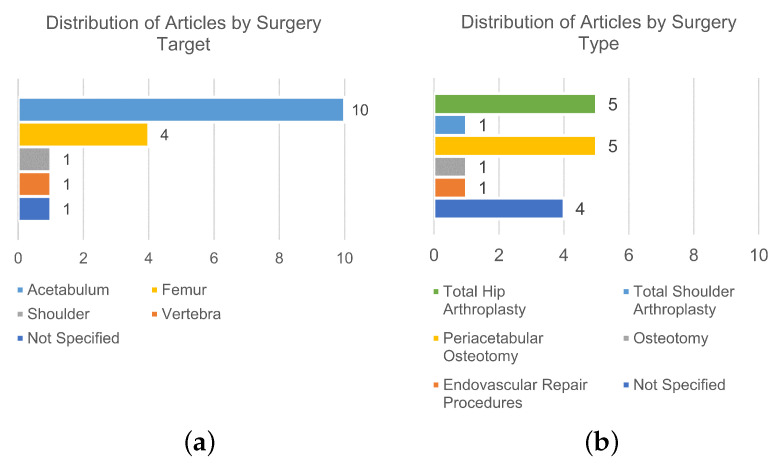
Analysis of the distribution of included studies based on the surgery target (**a**) and surgery type (**b**) for the image-based technologies.

Out of the 32 studies that met the eligibility criteria and were included in this review, 17 reported image-based technologies [53,54,55,56,57,58,59,60,61,62,63,64,65,66,67,68,69]. The distribution of the included studies based on the surgery target is illustrated in Figure 3a, while Figure 3b shows their distribution based on the surgery type.

From those 17 articles, 6 focused on measurements obtained from fluoroscopy images [53,54,55,56,57,58], 4 on measurements obtained from camera-based tracking [59,60,61,62], and 7 focused on measurements obtained from CT images [63,64,65,66,67,68,69]. As such, the results will be presented considering those subgroups and summarized in Table 2, Table 3, and Table 4, respectively.

#### 3.1.1. Fluoroscopy

Fluoroscopy devices, especially mobile ones, have been an integral part of orthopedic surgeries from the moment computer-assisted surgeries started being performed, as they provide real-time feedback of bone and surgical tool positions [71,72,73,74]. That capability makes fluoroscopy a potentially useful tool for angle measurements and is the focus of the articles presented below.

In 2008, Citak et al. [53] evaluated and compared the accuracy of navigation using the femoral neck axis with the greater trochanter, to determine femoral anteversion. Reference geometrics were placed marking important locations. Fluoroscopy images were taken, and the software calculated the angles, resulting in mean differences of $1.4^{\circ }$ and $0.3^{\circ }$ for the conventional and new techniques, respectively.

Troelsen [54], in 2009, developed a measuring device for intraoperative assessment of the acetabular index and center edge angle during acetabular reorientation in periacetabular osteotomy and assessed it against postoperative radiographs. The device is mounted bilaterally on the pelvis, and using fluoroscopy, angle measurements are obtained with adjustable measuring discs. Results showed that angle measurements differed less than $\pm 5^{\circ }$ from radiograph measurements. This device is based on work previously published by the same author [75].

Also developing a new method, Sidon and Steinberg [55], in 2012, evaluated the accuracy of a new computerized system capable of intraoperative planning and various measurements, using the developed software to measure relevant angles on panoramic images, compared it to a goniometer and a ruler and found no significant difference between the different techniques.

In 2013, Varnavas et al. [56] developed an automated method to compute the initial pose of tridimensional (3D) data in 2D-3D registration systems. Utilizing intraoperative fluoroscopy images, a generalized Hough Transform is applied using precomputed Digitally Reconstructed Radiographs, rendered from preoperative CT data. By applying a 3D rigid transformation, it pictures a single vertebra and can be extended to multiple vertebrae. The final registrations had an error of $\pm 2.5^{\circ }$ compared to a set of extrinsic parameters defining the 3D pose.

Also in 2013, Apivatthakakul et al. [57] compared the coronal femoral alignment between a new method of acquiring intraoperative panoramic images, developed in the study, and the conventional X-rays. In their work, an alignment grid was used to stitch fluoroscopy images, creating a panoramic image from where the coronal plane angulation could be derived. Results showed no significant difference between the different techniques.

Finally, in 2020, Dalbeth et al. [58] compared measurements of the angle of lateral opening and determined, using a radiopaque cup, a position assessment device imaged with fluoroscopy, with measurements obtained directly and by CT. The device should be placed within the acetabular component, and lateral fluoroscopy images were obtained. The digital calipers on the fluoroscopy unit were used to measure the length and derive the angles, finding no significant differences between the measurements.

#### 3.1.2. Camera Tracking Systems

Optical and electromagnetic tracking systems are two of the main technologies used by commercially available navigation systems in computer-assisted image-guided surgery [76]. The use of tracking-based augmented reality (AR) for surgical navigation has developed rapidly in recent years, and its utility in the surgery context is becoming apparent [26,77,78].

Tracking and navigation systems are outside of the scope of the present work; however, some articles that report on these systems reference the potential use of the technology for intraoperative angle measurement, which falls inside the scope of the study. The navigation systems are not interesting for the work, but the technology responsible for this angle measurement might be. With that in mind, the articles presented below reported on that measurement capability and therefore are included in this review.

Lin et al. [59], in 2008, evaluated the accuracy of an imageless computer navigation system on cadavers, comparing it to direct bone digitation. Trackers are rigidly fixed to the pelvis and femur, communicating via infrared signals to a camera system and a computer, where orientation is determined in real time. They found that the average difference between anteversion and abduction was $3.3\pm 3.5^{\circ }$ and $0.6\pm 3.7^{\circ }$, respectively.

In 2015, Chae et al. [60] developed an image-guided surgery system based on afocal optics, comparing it to a commercial optical tracker. It uses a marker with a lens and micro-engraved data-coded patterns that are captured by a camera on an afocal image, in which an orientation-tracking algorithm measures the angles. The afocal optical system provided accuracy equal to or better than the commercial, with an orientation error of $0.093^{\circ }$.

In 2018, Pflugi et al. [61] built upon their previous work, which was included in the non-image-based technologies, and developed a hybrid augmented marker-based navigation system for acetabular reorientation during periacetabular osteotomy, and compared it to an optical tracking system using a cadaveric study as well as a plastic bone study. A tracking unit is attached to the pelvis, while an augmented marker is attached to the acetabular fragment. The marker has an IMU to measure its orientation with a Kalman filter that fuses the marker tracking and IMU data. Mean absolute differences for inclination and anteversion were $1.34\pm 1.50^{\circ }$ and $1.21\pm 1.07^{\circ }$, respectively, for the cadaver study, and $1.63\pm 1.48^{\circ }$ and $1.55\pm 1.49^{\circ }$, respectively, for the plastic bone study.

Finally, in 2022, Hayashi et al. [62] compared the accuracy of 3D mini-optical navigation and an accelerometer-based portable navigation system for cup positioning, which is also studied in the next section, during a total hip arthroplasty in the supine position. A camera captures the movements of a tracker placed on the base unit. Three anatomical landmarks are registered, and the system provides real-time data. They obtained similar results for both technologies, comparing optical versus accelerometer-based with $2.8^{\circ }\pm 1.7^{\circ }$ versus $2.8^{\circ }\pm 1.9^{\circ }$ for inclination, and $2.6^{\circ }\pm 2.3^{\circ }$ versus $2.5^{\circ }\pm 1.9^{\circ }$ for ante version, respectively.

#### 3.1.3. Computed Tomography

Computed tomography has evolved from its introduction in 1972 to one of the most widely used imaging techniques [79,80,81,82]. Its use has been recommended intraoperatively for the ability to provide real-time feedback [83], sometimes coupled with the previously described camera tracking systems, and the articles presented below show its application for angle measurement.

In 1998, DiGioia et al. [63] presented an image-guided navigation system, HipNav, that continuously measures the pelvic location and tracks relative implant alignment intraoperatively. It comprises three components: a preoperative planner based on CT data, a hip RoM simulator, and intraoperative tracking using an optical tracking camera. The preoperative data are matched with the intraoperative position allowing for the continuous monitoring of the pelvis orientation. The system was successfully used in an operating room with minimal impact on the surgical routine.

In 2007, Armiger et al. [64] proposed a technique for computationally measuring the radiological angles from a joint contact surface model, segmented from CT-scan images, and compared it with manual measurements. Their developed Lunate–Trace algorithm was applied to CT scans, resulting in minor discrepancies between the manual and computerized techniques. The measurement error for the proposed computer method was $-1.30\pm 3.30^{\circ }$.

Also in 2007, Nguyen et al. [65] developed and evaluated the computer-assisted glenoid implantation technique to achieve more accurate and reliable placement of the glenoid component and compared it to CT results. Using surface modeling software, 3D models are created on CT slice data, and a validated electromagnetic tracking system calculates the angles. Statistically significant differences were found in the $1^{\circ }$ range, and the authors conclude it is unlikely to be clinically significant.

Hawi et al. [66], in 2014, reported the accuracy of using ISO-C 3D for the measurement of femoral ante torsion intraoperatively, comparing it with conventional multi-slice CT. ISO-C 3D combines a mobile image intensifier with CT images and uses a previously established method for calculating angles [70]. No significant difference between the different techniques was found. Additionally, the mean time to perform a scan at 9±3 min and the mean time to measure ante torsion at 8±2 min.

Murphy et al. [67], in 2015, presented and validated a computer-navigated system for performing periacetabular osteotomy to treat developmental dysplasia of the hip. They developed the Biomechanical Guidance System, an optoelectronic tracking system, which consisted of a navigation camera, a patient-mounted reference, and a tracker that can be used in tools or limbs, which the software uses to compute the radiological measurements based on 3D models created from preoperative CT images. It was tested in two cases using fiducial markers, computing measures that differed from measured fiducial transformations by $1.0^{\circ }$ and $2.2^{\circ }$ in rotations, respectively.

In 2018, Ogawa et al. [68] developed an acetabular cup placement device using augmented reality, AR-HIP, capable of intraoperative measurements and compared it to a goniometer. The device uses CT images and multiplanar reconstruction to obtain 3D coordinate points and, by aligning the superimposed image on the view of the actual cup, calculates anteversion and inclination angles. AR-HIP was significantly more accurate in measuring anteversion ($2.7^{\circ }$ versus $6.8^{\circ }$) and not significantly different in measuring inclination ($2.1^{\circ }$ versus $2.6^{\circ }$).

Finally, also in 2018, De Raedt et al. [69] validated the use of computer navigation with a minimally invasive trans sartorial approach in periacetabular osteotomy, compared to the manual measurements performed using a custom-developed application. The team had elements from the works by Armiger et al. [64] and Murphy et al. [67] using the Lunate–Trace algorithm to obtain data from CT images obtained preoperatively. The Biomechanical Guidance System was used to calculate the angles intraoperatively based on the data retrieved, reporting angle measurements showing good agreement with manual angle measurements.

### 3.2. Non-Image-Based Technologies

For the purposes of this review, non-image-based technologies refer to those that do not depend on images, or data originating from images, to calculate the relevant angle measurements. Similarly to the previous section, some articles mentioned more than one technology as a comparison, nonetheless, these articles are grouped considering the technology that is the main focus of the study.

**Figure 4:**
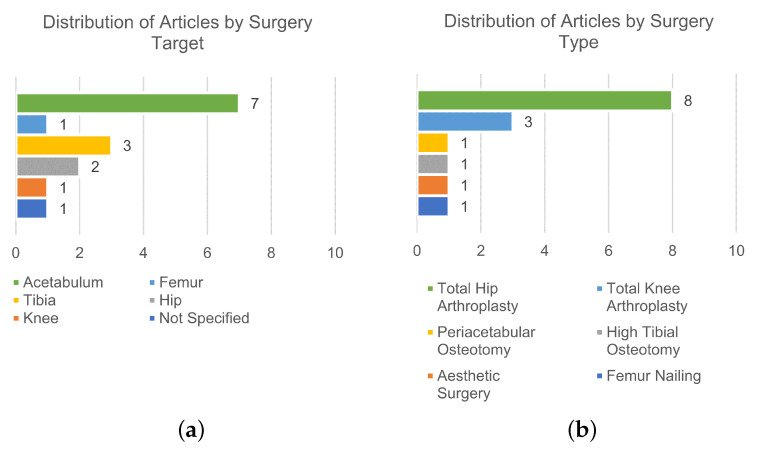
Analysis of the distribution of included studies based on the surgery target (**a**) and surgery type (**b**) for the non-image-based technologies.

Of the 32 articles in the study, 15 reported on technology that is non-image-based [84,85,86,87,88,89,90,91,92,93,94,95,96,97,98] and will be included in this section. The distribution of the included studies based on the surgery target is illustrated in Figure 4a, while Figure 4b shows their distribution based on the surgery type.

Out of those 15 articles, 6 focused on measurements from manual instruments [84,85,86,87,88,89], and 9 focused on inertial-based instruments [90,91,92,93,94,95,96,97,98], encompassing IMU-, accelerometer-, gyroscope-, and magnetometer-based instruments. The results will be presented considering those subgroups and summarized in Table 5 and Table 6, respectively.

#### 3.2.1. Manual Instruments

In a time where a lot of the research is concentrated on computer-assisted surgery, traditional surgical instruments are still used in highly active roles in almost all surgical procedures. Since the surgeon has complete control over the instruments, they can be used with confidence for most of the necessary manipulation tasks [99]. Different manual instruments exist capable of measuring angles, and these can be found in the articles below.

Vendittoli et al. [84], in 2002, evaluated the potential benefit of an inclinometer during vertical acetabular implant positioning by comparing it with visuospatial perception. The inclinometer is designed for attachment to most acetabular insertion rods by hand-tightening a single screw, scaled at $2^{\circ }$ intervals from $0^{\circ }$ to $70^{\circ }$, and its position measurement is $42.2\pm 3.8^{\circ }$ compared with the more uncertain perception measurement of $44.4\pm 11.4^{\circ }$.

In 2012, Sykes et al. [85] developed and evaluated a novel mechanical device, the closed-tube inclinometer, for measuring operative acetabular inclination, comparing it to free-hand and the mechanical alignment guide. The closed-tube inclinometer is used in conjunction with the transverse acetabular ligament to aid cup orientation, and two trials were performed where the inclinometer showed better results when compared to the other techniques.

In 2013, McGann et al. [86] developed and validated an intraoperative device to precisely measure knee flexion and extension. This knee goniometer consisted of a digital level mounted to a base that rigidly attaches two needles to be pushed through the soft tissue. Their results showed a systematic error ranging from $-9.1^{\circ }$ to $3^{\circ }$ and the measurement error was $1.5\pm 1^{\circ }$.

Meermans et al. [87], in 2015, investigated whether the use of a digital protractor to measure the operative inclination angle could improve the positioning of the acetabular component in relation to a “safe zone”, comparing it with free hand placement. A digital inclinometer was used to measure the operative inclination angle and significantly reduced the number of acetabular component inclination outliers compared with freehand positioning.

In 2020, Jeong and Park [88] devised a specific ruler for perioral measurements. The ruler has different colored lines for length and angle measurements and a reference point for its correct positioning. It is composed of a rectangular body (10×6 cm in size) and two arms (3×0.5 cm in size), with red lines representing angle intervals of $3^{\circ }$, and it was validated during a corner mouth lift procedure.

Finally, Chuaychoosakoon et al. [89], also in 2020, evaluated if a four-reference Kirchner wire (K-wire) technique can reliably assess actual alignment correction during surgery after determination of the desired corrective angle. The technique consists of two coronal K-wires placed at an angle measured using a goniometer and two sagittal K-wires placed parallel to each other to ensure the tibial slope is maintained. No statistically significant differences were found between the desired amount of alignment correction and the corrections achieved through the use of the four-reference K-wire technique intraoperatively.

#### 3.2.2. Inertial-Based Instruments

Inertial-based sensors, such as IMUs, consisting of accelerometers and gyroscopes, and sometimes with the addition of magnetometers, are widely used in different applications [100,101,102,103,104]. Mainly used to obtain data on velocity, orientation and gravity force, their applications range from robotics to navigation and have been applied to medicine [105].

The articles presented below used inertial-based techniques to obtain orientations and applied them in an intraoperative context.

In 2012, Peters et al. [90] developed a system that uses the accelerometer and camera function of the iPhone to improve acetabular cup placement, and, using an indicator phone application, all cups were placed within a narrow range in the safe zone, and less than 5% differences were found comparing pre-, intra-, and postoperative inclinations.

In 2013, Hawi et al. [91] investigated and validated the accuracy of a new method based on positional technology integrated into smartphones and compared to traditional methods, CT and surgical navigation. A standard goniometer application for smartphones, based on the integrated gyroscope, was used, and a device was designed to fixate the smartphone to the patient. They found a fair or good correlation between the new method and the traditional ones in all scenarios with no statistically significant differences.

Nam et al. [92], in 2014, compared the tibial component alignment obtained using an accelerometer-based navigation device versus extramedullary alignment guides. They used the KneeAlign, composed of a disposable display console and a reference sensor, both containing a three-axial accelerometer. Results showed that 95.7% of tibial components were within $2^{\circ }$ of perpendicular to the tibial mechanical axis and 95.0% were within $2^{\circ }$ of a $3^{\circ }$ slope, for the device, compared with 68.1% and 72.1% for extramedullary guide, respectively.

Pflugi et al. [93], in 2016, evaluated an inertial sensor-based surgical navigation solution for periacetabular osteotomy surgery by using two IMUs with a variation of the Kalman filter for data fusion. No statistically significant difference was found in the measurement of acetabular component reorientation. They later incorporated augmented reality in the study included in the previous section [61].

In 2018, Chen et al. [94] proposed and validated a three-part measurement system composed of initial attitude measurement, real-time attitude measurement, and acetabular cup measurement. Each system is based on a nine degrees of freedom (DOF) IMU with a quaternion-based extended Kalman filter. Results found the root mean square error of attitude and acetabular orientation are less than $1.6^{\circ }$ and $3^{\circ }$ with uncertainty of less than $0.22^{\circ }$ and $0.17^{\circ }$, respectively.

In 2019, Tang et al. [95] developed an IMU-based hip smart trial system, the IMUHST, for intraoperative monitoring of hip posture. A nine DOF IMU with Kalman filtering computes the rotation angle. When measuring in the three axes, the mean absolute errors found were: $2.5\pm 4.9^{\circ }$ for flexion/extension; $2.5\pm 4.4^{\circ }$ for adduction/abduction; and $1.0\pm 2.0^{\circ }$ for internal/external rotation.

Kamenaga et al. [96], in 2019, and, Takada et al. [97], in 2020, used the HipAlign, built on the same principles as the KneeAlign [92], to assess the accuracy of cup orientation. The first found similar errors between the device and a manual goniometer, while the second found an average absolute error when compared to postoperative CT, of $2.6\pm 2.7^{\circ }$ for inclination and $2.8\pm 2.7^{\circ }$ for anteversion.

Finally, in 2022, Kokko et al. [98] assessed a novel gyroscope-based instrument for intraoperative validation of tibia coronal plane alignment. A prototype was outfitted with an IMU evaluation board to estimate the alignment using the gyroscope output and custom MATLAB^®^ scripts (MathWorks, Natick, MA, USA). The average accuracy was estimated to be within $\pm 1^{\circ }$.

## 4. Discussion

### 4.1. Interpretation and Implications of the Results

The 32 articles included in this review made use of different technologies, including image-based and non-image-based, for intraoperative angle measurement. Overall, all articles reported good results for the techniques in study, whether in comparison with a traditional technique or by determining the measurement error of the technology.

Image-based technologies, based on fluoroscopy, CT, and camera tracking, are commonly used intraoperatively since multiple available guidance and navigation systems use them [36,52,106,107,108]. The major advantage of image-based techniques is their reliable accuracy, as all of the included studies showed, as well as the capability of 3D tracking, particularly when used with a tracking system [65,109]. This is in part explained by the high accuracy of the marker-based camera tracking systems, which typically present better results when compared to other motion capture systems (e.g., markerless optical and inertial motion capture systems) [110,111].

However, there are some drawbacks associated with this form of measurement. Firstly, in the case of fluoroscopy and CT-based technologies, obtaining the images means increased ionizing radiation exposure and its associated risks [112], the use of specialized equipment, and the need for consistent positioning of the subject and the instrumentation machine, which, in turn, creates difficulties with the existence of a learning curve to master these techniques and added surgery time [45]. Hawi et al. [66] reported a mean time of 9±3 min to obtain the images, and a mean time of 8±2 min to measure ante torsion, which is consistent with the previously mentioned increase in procedure time. This added time is even more note-worthy if the imaging tool is not associated with a guidance system, which in turn carries high costs, as it will not be optimized for surgical procedures and will usually require additional personnel to handle the technology. Secondly, despite its reported high accuracy and possibility of acquiring the segment’s kinematics in real-time [113], camera tracking, specifically, also has an added concern with the accurate placement of the markers in the correct landmarks, creating another level of difficulty that may influence the resulting measurements [114,115,116], either by misplacement of the markers or misidentification of the landmarks by the surgeon. Additionally, if the position of the targets shifts during a surgical procedure, or an obstacle blocks the camera’s line-of-sight, the measuring process may be compromised for the remainder of said procedure [116,117]. Added surgery time is also a concern in this modality as the preparation of the equipment and marker placement can be quite time-consuming [118]. Finally, there are also increased costs associated with the use of image-based technologies. The equipment itself and the training necessary to use it, as well as the added surgery time, come with added costs that one needs to be aware of before implementing these technologies [45]. Nevertheless, the use of tracking systems together with 3D representations of the segments could have a deep impact on both the surgical team and the patient, as it can result in a reduction of the operation time and radiation exposure, two of the major limitations of this technology [119]. Moreover, in the scope of implementing image-based technologies, despite requiring a considerable investment of resources, the capabilities they offer justify their use primarily for complex operations that depend on those capabilities [44].

Non-image-based technologies, namely manual instruments and inertial-based instruments are as much of a staple in operating rooms, if not more so in the case of manual instruments, and usually demand lower costs. Since they are imageless technologies, there is no exposure to radiation. Additionally, these systems are usually easy to handle. However, a potential disadvantage is the accuracy when compared with image-based technologies. There have been cases reported where non-image-based techniques are not as accurate when compared to image-based techniques [120,121]. Nonetheless, the studies included in this review all showed great accuracy when compared to image-based technologies, which is in line with relevant works comparing imageless and traditional technologies [122,123].

For the case of inertial-based technologies specifically, advantages include being low-cost (depending on the components used), easy to use, and accurate; can provide feedback in real-time; and allow for 3D calculations without the need for image segmentation or surgeon intervention, as was shown in Pflugi et al. [93]. However, they also come with drawbacks as there is a learning curve associated with their use when compared with manual instruments. Additionally, the use of this technology can be susceptible to interference, especially if it uses a magnetometer [61] as magnetic material not accounted for in the calibration stage will alter the output of the magnetometer [124,125]. Some care for the positioning of the sensors is also needed since an abrupt position change may also cause measurement errors. Inertial sensors require frequent calibration for accurate measurements [61,126], and in the case those calibrations are lost, the measuring process, and consequently the surgical procedure, may be compromised. Even when calibrated, orientation estimation requires some processing of the sensor data, usually in the form of a sensor fusion algorithm, to obtain useful measurements as noise, bias, and drift (mainly from the gyroscope) are well-reported issues in the literature [127,128]. Furthermore, inertial-based sensors typically present good accuracy in angle measurements. However, if the procedure also requires translation assessments, the advantage of this use in comparison with camera-based tracking systems is reduced, as they typically require additional methods to achieve comparable accuracy outcomes [111,129,130,131,132]. Nevertheless, novel algorithms have been proposed to better correct the sensor alignment with the body segment [133,134,135,136] or to compensate for some of the issues previously described [137,138,139], improving the overall accuracy and reliability of the outcomes.

Manual instruments are accurate, practical, easily sterilized, and have reduced costs, but are often dependent on the surgeon’s visuospatial perception [84], which is one of the main reasons the other technologies exist in the first place. As in many other fields, the trend points towards manual instruments being replaced by more advanced technologies since surgical instruments are intimately linked to technology [140]; however, their use is still justified since there may be contexts (e.g., in cases where the location of interest makes any other technology unusable) where they are the only available tool for angle measurement.

Hence, implementing non-image-based technologies is particularly useful in simple operations, where the capabilities of image-based technologies are not required, and therefore their drawbacks are not justifiable. Moreover, concerns related to radiation exposure or the impact on the patient’s health due to the utilized equipment further underscore the merit of opting for non-image-based approaches.

In clinical practice, ensuring intraoperative precision in measuring osteotomy angles is of paramount importance [43,44]. Orthopedic surgeons are mostly acquainted with the use of manual devices that are inexpensive and easy to use; however, their accuracy is still far from precise. In recent years, intraoperative navigation systems have been introduced in joint replacement, providing accurate implantation of the prosthesis. These are expensive devices with a main goal: to provide perfect alignment of the prostheses. However, there is limited academic literature on this subject when focusing on the intraoperative measurement of corrective osteotomies, especially for angular or rotational deformities. The majority of existing studies are centered around preoperative planning, lacking comprehensive perioperative control, and only 8 of the 32 studies identified related to osteotomy correction intraoperatively [54,55,61,64,67,69,89,93], with the focus mostly on periacetabular osteotomies, representing six of those studies [54,61,64,67,69,93]. Consequently, many surgeons still rely on conventional goniometers or pre-set wedges to gauge osteotomy corrections, and assessing rotational osteotomy corrections poses an even more formidable challenge. This highlights the potential of both image-based and inertial-based technologies to significantly enhance the quantitative evaluation of angles between segments or bones, as these systems typically offer higher levels of accuracy compared to the surgeon’s visuospatial perception alone [84]. Furthermore, systems equipped with orientation-tracking technology, such as inertial or camera tracking systems, empower surgeons to execute 3D angular deformity corrections, mitigating the limitations often associated with 2D technologies like radiographic imaging [36,119,141,142]. When conducting a comparative analysis between image-based and non-image-based technologies, the central point revolves around the requisite level of accuracy (when radiation exposure is not a factor). While non-image-based technologies have been shown capable of great accuracy, there are conflicting findings in the literature on the topic [120,121]. Consequently, when a procedure requires the highest level of accuracy, opting for image-based technologies is advisable. Conversely, when the accuracy requirement is not as stringent, non-image-based measurement systems emerge as a more economical, user-friendly, radiation-free, and time-efficient alternative while also being capable of providing 3D feedback in real time. When all factors are considered, the specific procedural requirements should serve as the principal determinant in the selection of technology.

### 4.2. Future Trends

In the search for ideal commercial solutions, the continuous evolution of new technologies, as well as novel applications for existing ones, is expected to play a pivotal role in shaping the future of intraoperative angle measurement. As research on this topic intensifies, innovative methods are likely to surface, addressing current limitations and introducing more efficient and accurate solutions. Over the short-run horizon, inertial-based sensors are a potential focus of development, mainly due to their cost-efficiency and ease of use. Currently, there is a research focus aimed at integrating this technology into diverse surgical solutions [143,144], as well as improving its performance by developing new algorithms for angular estimation [137,138,139,145]. The progressive integration of navigation systems in surgical procedures is poised to bring about transformative changes for intraoperative angle measurement in the long run [146,147]. These technologies already have the capabilities required for intraoperative angular measurement, and the ongoing developments will improve their cost-effectiveness and minimize the learning curves associated with their use. Among these, markerless systems, namely inertial- or camera-based tracking systems, are gaining prominence as a trending technology as they circumvent radiation exposure and mitigate the limitations associated with marker placement while ensuring high-accuracy outcomes [61,119,148,149,150,151]. In this scope, several methodologies have been proposed to improve the accuracy of these systems, including biomechanical models and advanced algorithms, to correct or better predict the segment orientations [152,153,154,155]. As technology advances, it is anticipated that these novel approaches will gradually replace the need for medical imaging during surgery, thereby enhancing the surgeon’s spatial awareness of anatomical structures. Ultimately, this evolution is expected to deliver tangible benefits to both patients and surgical teams. Nonetheless, the role of preoperative image-based technologies will remain essential, both to assist the surgical team in operation planning and to provide reliable representations of the segments that serve as inputs for these intraoperative angle measurement systems. Future trends should focus on the continual refinement of technologies, offering reliable, cost-efficient, autonomous, and radiation-free solutions for accurate intraoperative angle measurements.

### 4.3. Limitations

This systematic review followed the PRISMA statement, and the methodology was followed; however, some limitations were found compared with the suggested procedure. Firstly, the review lacks in the assessment of the results, both in quality and bias. Due to the type of review presented, where the goal was to provide an overview of the available techniques and their reported performance rather than conducting a meta-analysis, the non-inclusion of this assessment does not influence the conclusions reached. Nonetheless, future work may improve on the present work by including such an assessment. Additionally, the included studies all reported great accuracy in the results, which may be associated with the type of article retrieved. This may be a source of bias since the articles showcase the development of a device or method and are therefore much more likely to report positive results. Attempted solutions that do not show good performance are left unpublished.

Nevertheless, to our knowledge, this is the first review on this topic. It gathered all the relevant works, from the searched databases, by following a detailed and reproducible methodology.

## 5. Conclusions

The focus of this work was to present a comprehensive review of the available intraoperative angle measurement technologies and their performance. For that purpose, PubMed, Scopus, Web of Science, Cochrane Library, and IEEE Xplore databases were searched to look for studies and, after applying eligibility criteria and analyzing the references of included studies, 32 total studies were included in this review. The articles were grouped considering the use (or not) of images in the technology, with 17 articles reporting on image-based technology (of which 6 are fluoroscopy, 4 are camera-based tracking, and 7 are CT-based) and 15 non-image-based techniques (of which 6 are manual instruments and 9 are inertial-based instruments).

Overall, all the studies reported good results for the techniques used in the study, with reliable accuracy and low measurement errors. Image-based techniques are commonly used intraoperatively, and they provide reliable accuracy and 3D tracking capabilities. However, they also have associated drawbacks, such as increased radiation exposure, specialized equipment requirements, a learning curve, added surgery time, and increased costs. Non-image-based techniques are imageless technologies that are usually easy to handle and have lower costs. They also provide great accuracy, and although they may not be as accurate as image-based techniques in some cases, the results in this review showed great accuracy across all reports. The drawbacks associated with these technologies include dependency on the surgeon’s visuospatial perception and, in the case of inertial-based technologies, susceptibility to interferences and uncalibrated measurements, and a learning curve.

The selection of using one technology over the other is contingent on the specific context in which this technology will be applied, with the determination resting upon an evaluation of whether the benefits surpass the drawbacks within that particular context. The trajectory of future trends in this field is poised for rapid evolution as research and development endeavors intensify. The promising short-term focus on inertial-based sensors and the ongoing integration of navigation systems underscore a trajectory toward transformative changes. The increasing prominence of markerless camera-based tracking further highlights the shift towards reduced radiation solutions with high accuracy, to ensure optimal outcomes for patients and facilitate the continual progress of medical science.

Ultimately, this work succeeded in presenting a comprehensive review of the available intraoperative angle measurement technologies, their performance, advantages and disadvantages. It is a useful guidance tool for surgeons needing to obtain angle measurements in a surgical context, in addition to facilitating the development of new technologies aiming to handle the limitations identified for the different existing methods.

## Figures and Tables

**Table 1:** Initial keyword groups.

Surgery	Angle	Measurement	Intraoperative
surgical operation	orientation	determination	perioperative
surgical procedure	pose	calculation	in-surgery
surgical intervention	angular position	computation	intrasurgical
surgical technique	angular displacement	assessment	operating room
osteotomy		evaluation	real-time
		estimation	
		mensuration	
		quantification	
		valuation	

**Table 2:** Summary of articles reporting fluoroscopy-based angle measurement.

First Author (Year)	Technology	Target	Main Results
Citak (2008) [53]	Fluoroscopy images Software calculated the angles	Femoral anteversion	Mean differences of 1.4° and 0.3° on the conventional and new techniques, respectively
Troelsen (2009) [54]	Fluoroscopy images Measurements obtained by a device with adjustable measuring discs	Acetabular reorientation during periacetabular osteotomy	Angle measurements differed less than ±5° from radiograph measurements
Sidon (2012) [55]	Panoramic view image from fluoroscopy images Software measures the angles	Varus or valgus angles during femur osteotomy	No significant difference between the different techniques
Varnavas (2013) [56]	Generalized Hough Transform to fluoroscopy images Maximize the Gradient Difference Similarity Measure to establish initial pose	Endovascular procedures	Final registration within ±2.5° of ground truth
Apivatthakakul (2013) [57]	Alignment grid stitches fluoroscopy images Obtain the coronal plane angulation	Coronal plane alignment of femur fractures	No significant difference between the different techniques
Dalbeth (2020) [58]	Fluoroscopy images Digital calipers derive the angles	Acetabular component position in total hip arthroplasty in dogs	No significant difference between the different techniques

**Table 3:** Summary of articles reporting camera-based tracking angle measurement.

First Author (Year)	Technology	Target	Main Results
Lin (2008) [59]	Rigidly fixed trackers, communicate via infra-red signals with camera system and computer Orientation is determined in real-time	Acetabular cup orientation during total hip arthroplasty	Average difference for anteversion and abduction were 3.3 ± 3.5° and 0.6 ± 3.7°, respectively
Chae (2015) [60]	Marker with a lens and micro-engraved data-coded patterns captured by a camera on an afocal image Orientation-tracking algorithm measures the angles	Position and orientation during surgical navigation	The afocal optical system provided accuracy equal to or better Orientation error of 0.093°
Pflugi (2018) [61]	Tracking unit and augmented marker with an IMU Kalman filter fuses the marker tracking and IMU data	Acetabular orientation during periacetabular osteotomy	Mean absolute differences for inclination and anteversion were 1.34 ± 1.50° and 1.21 ± 1.07°, respectively, for the cadaver study 1.63 ± 1.48° and 1.55 ± 1.49°, respectively, for the plastic bone study
Hayashi (2022) [62]	Camera captures the movements of a tracker placed on the base unit Three anatomical landmarks are registered System provides real-time data	Cup positioning during a total hip arthroplasty	Similar results comparing optical versus accelerometer-based: 2.8° ± 1.7° versus 2.8° ± 1.9° for inclination 2.6° ± 2.3° vs. 2.5° ± 1.9° for ante version, respectively

**Table 4:** Summary of articles reporting CT-based angle measurement.

First Author (Year)	Technology	Target	Main Results
DiGioia (1998) [63]	CT preoperative data matched with the intraoperative tracking data	Acetabular alignment during total hip replacement	The system was successfully used in operating room with minimal impact on surgical routine
Armiger (2007) [64]	CT scans Lunate–Trace algorithm to CT images	Joint alignment during periacetabular osteotomy	Minor discrepancies between manual and computerized technique. The measurement error for the proposed computer method was −1.30 ± 3.30°
Nguyen (2007) [65]	3D models created from CT slice data Tracking system obtains angles	Placement of the glenoid component during total shoulder arthroplasty	Statistically significant differences were found, however, in the 1° range The authors assume it is unlikely to be clinically significant
Hawi (2014) [66]	Mobile image intensifier with CT (ISO-C 3D) images Previously established method for calculating angles [70]	Femoral antetorsion	No significant difference between different techniques. Mean time to perform scan: 9 ± 3 min. Mean time to measure ante torsion: 8 ± 2 min
Murphy (2015) [67]	3D models created from preoperative CT images Biomechanical Guidance System, camera-based tracking	Characterization of the acetabulum during periacetabular osteotomy	Computed measures differed from measured fiducial transformations by 1.0° and 2.2° in rotations, in two cases
Ogawa (2018) [68]	CT images and multiplanar reconstruction obtains 3D coordinate points Alignes the superimposed image on the view of actual cup	Acetabular cup placement during total hip arthroplasty	AR-HIP was significantly more accurate measuring anteversion 2.7° versus 6.8° Not significantly different measuring inclination 2.1° versus 2.6°
De Raedt (2018) [69]	CT data obtained preoperatively Lunate–Trace algorithm and Biomechanical Guidance System with CT data	Characterization of the acetabulum during periacetabular osteotomy	Intra-operative reported angle measurements showed good agreement with manual angle measurements

**Table 5:** Summary of articles reporting manual instrument-based angle measurement.

First Author (Year)	Technology	Target	Main Results
Vendittoli (2002) [84]	Inclinometer with acetabular insertion rods by hand-tightening a single screw Scaled at 2° intervals from 0° to 70°	Vertical acetabular positioning during total hip arthroplasty	Position precision for the inclinometer is 42.2 ± 3.8° compared with 44.4 ± 11.4°
Sykes (2012) [85]	The closed-tube inclinometer used with the transverse acetabular ligament	Acetabular inclination during total hip arthroplasty in the lateral decubitus position	Two trials were performed for three techniques (freehand, mechanical guide and inclinometer): In the first one, the mean errors were: 5.2 ± 4.3°, 3.6 ± 3.9°, 0.5 ± 0.4° In the second they were: 6.2 ± 4.2°, 3.8 ± 3.3°, 0.6 ± 0.5°
McGann (2013) [86]	Knee goniometer: digital level mounted to a base attached to two needles	Knee flexion and extension during total knee arthroplasty	Systematic error ranged from −9.1° to 3° Measurement error was 1.5 ± 1°
Meermans (2015) [87]	Digital inclinometer	Inclination of the acetabular component during total hip arthroplasty	Significantly reduced the number of acetabular component inclination outliers compared with freehand positioning
Jeong (2020) [88]	Ruler with differently colored lines	Perioral ruler for routine aesthetic surgery	A woman received corner mouth lift where the angle of the corner of the mouth measured from the stomion changed from −6° to 3°
Chuaychoosakoon (2020) [89]	Two coronal K-wires placed at an angle measured using a goniometer Two sagittal K-wires placed parallel to each other to ensure tibial slope is maintained	Coronal alignment correction during opening-wedge valgus high tibial osteotomy	No statistically significant differences were found between the desired amount of alignment correction and the corrections achieved

**Table 6:** Summary of articles reporting inertial-based instrument angle measurement.

First Author (Year)	Technology	Target	Main Results
Peters (2012) [90]	Level indicator and a protractor application using accelerometer and the camera	Acetabular cup orientation during total hip arthroplasty	All cups were placed within a narrow range in the safe zone and less than 5% difference between pre-, intra- and postoperative inclinations
Hawi (2013) [91]	Standard goniometer application for smartphones using gyroscope	Femoral antetorsion during femoral nailing	Found a fair or good correlation between the new method and the traditional ones in all scenarios with no statistically significant differences
Nam (2014) [92]	KneeAlign: disposable display console and a reference sensor both containing a 3-axial accelerometer	Tibial alignment during total knee arthroplasty	95.7% of tibial components were within 2° of perpendicular to the tibial mechanical axis and 95.0% were within 2° of a 3° slope, for the device, compared with 68.1% and 72.1% for the extramedullary guide
Pflugi (2016) [93]	Two IMUs fuse data using a variation of the Kalman filter	Orientation of the acetabular fragment during periacetabular osteotomy	No statistically significant difference was found on the measurement of acetabular component reorientation
Chen (2018) [94]	9-DOF IMU with a quaternion-based extended Kalman filter	Implantation angles during total hip replacement	RMSE of attitude and acetabular orientation are less than 1.6° and 3° with uncertainty of less than 0.22° and 0.17°, respectively
Tang (2019) [95]	9-DOF IMU with Kalman filtering	Hip posture during total hip arthroplasty	Mean absolute errors in measuring in the 3 axes: 2.5 ± 4.9° for flexion/extension; 2.5 ± 4.4° for adduction/abduction; and 1.0 ± 2.0° for internal/external rotation.
Kamenaga (2019) [96]	HipAlign: accelerometer-based device with a disposable computer display unit and a reference sensor	Acetabular cup orientation during total hip arthroplasty in the supine position	The average absolute error in measurement compared with postoperative CT navigation: 2.6 ± 2.7° for inclination 2.8 ± 2.7° for anteversion
Takada (2020) [97]	HipAlign: accelerometer-based device with a disposable computer display unit and a reference sensor	Acetabular cup orientation during total hip arthroplasty in supine position	Achieved similar errors between methods: with the HipAlign registering 3.3 ± 2.7° and 3.8 ± 3.4° and the manual goniometer 3.0 ± 2.5° and 6.0 ± 3.7°
Kokko (2022) [98]	ST LSM6DS3 IMU evaluation board using the gyroscope output and custom MATLAB^®^ scripts	Tibia coronal plane alignment during total knee arthroplasty	Average accuracy was estimated within ±1°

## Data Availability

No new data were created or analyzed in this study. Data sharing is not applicable to this article.

## Supplementary Materials

The following supporting information can be downloaded at: https://www.mdpi.com/article/10.3390/s24051613/s1, Table S1: Complete search strategy for the five electronic databases searched, with the results for every keyword, phrase and combination of groups using Boolean operators (*—refers to a truncated word).
