# Supplementary material for: Intraoperative Angle Measurement of Anatomical Structures: A Systematic Review
Source: Sensors (Basel). 2024 Mar 1;24(5):1613. doi: 10.3390/s24051613 (PMC10934548; doi:10.3390/s24051613)
Supplement: Supplementary file 1 [file sensors-24-01613-s001.zip › sensors-2873664-supplementary.pdf]

Review

Intraoperative Angle Measurement of Anatomical Structures: A Systematic Review

Table S1: Complete search strategy for the five electronic databases searched, with the results for every keyword, phrase and combination of groups using Boolean operators (* - refers to a truncated word).

Search number	Terms	Results				
		PubMed (MEDLINE)	Cochrane Library	Web of Science	Scopus	IEEE Xplore
#1	surgery	1,460,164	257,273	1,457,962	2,667,953	27,686
#2	surgical operation	4,290	16,603	90,587	271,703	1,847
#3	surgical procedure	50,644	27,971	247,055	833,295	3,413
#4	surgical intervention	62,834	28,176	125,082	202,510	1,218
#5	surgical technique	44,401	19,884	181,894	640,022	2,444
#6	osteotomy	34,203	2,333	38,152	67,206	121
#7	#1 OR #2 OR #3 OR #4 OR #5 OR #6	1,568,781	275,661	1,684,825	3,152,444	28,889
#8	angle measure*"	6,534	2	25,005	36,313	2,520
#9	angle determin*"	137	0	1,019	1,837	200
#10	angle calculate*"	0	0	228	743	35
#11	angle comput*"	16	0	216	500	159
#12	angle assess*"	145	0	222	279	22
#13	angle evaluat*"	71	0	184	295	33
#14	angle estimate*"	73	0	465	750	261
#15	angle mensurat*"	0	0	0	0	0
#16	angle quantif*"	6	0	34	52	2
#17	angle valuat*"	0	0	0	1	0
#18	orientation measure*"	302	2	1,565	2,296	351
#19	orientation determin*"	161	0	849	1,260	136
#20	orientation calculate*"	0	0	46	105	7
#21	orientation comput*"	8	0	84	159	81
#22	orientation assess*"	52	0	107	158	5
#23	orientation evaluat*"	24	0	95	165	11
#24	orientation estimate*"	84	0	357	500	177
#25	orientation mensurat*"	0	0	1	1	0
#26	orientation quantif*"	10	0	34	42	6
#27	orientation valuat*"	0	0	1	0	0
#28	"pose measure*"	31	0	587	908	319
#29	"pose determin*"	25	0	307	430	177
#30	"pose calculate*"	0	0	8	21	4
#31	"pose comput*"	0	0	150	260	113
#32	"pose assess*"	0	0	15	21	5
#33	"pose evaluat*"	11	0	45	64	26
#34	"pose estimate*"	40	0	925	1170	632
#35	"pose mensurat*"	0	0	0	0	1
#36	"pose quantif*"	0	0	0	0	0
#37	"pose valuat*"	0	0	0	1	0
#38	angular position measure*"	7	0	87	146	79

Continued on next page

Table S1: Complete search strategy for the five electronic databases searched, with the results for every keyword, phrase and combination of groups using Boolean operators (* - refers to a truncated word). (Continued)

Search number	Terms	Results				
		PubMed (MEDLINE)	Cochrane Library	Web of Science	Scopus	IEEE Xplore
#39	angular position determin*"	0	0	7	14	5
#40	angular position calculate*"	0	0	1	3	1
#41	angular position comput*"	0	0	1	5	2
#42	angular position assess*"	0	0	0	0	1
#43	angular position evaluat*"	0	0	2	1	0
#44	angular position estimate*"	0	0	4	8	4
#45	angular position mensurat*"	0	0	0	0	0
#46	angular position quantif*"	0	0	0	0	0
#47	angular position valuat*"	0	0	0	0	0
#48	angular displacement measure*"	26	0	101	171	52
#49	angular displacement determin*"	0	0	7	9	2
#50	angular displacement calculate*"	0	0	1	2	0
#51	angular displacement comput*"	0	0	0	0	4
#52	angular displacement assess*"	0	0	0	0	0
#53	angular displacement evaluat*"	0	0	2	2	0
#54	angular displacement estimate*"	2	0	2	2	0
#55	angular displacement mensurat*"	0	0	0	0	0
#56	angular displacement quantif*"	0	0	0	0	0
#57	angular displacement valuat*"	0	0	0	0	0
#58	#8 OR #9 OR (...) OR #56 OR #57	7,671	4	32,384	48,058	6,451
#59	intraoperative	162,974	31,344	141,601	232,347	1,331
#60	perioperative	126,676	23,356	116,252	163,360	151
#61	in-surgery	21,620	1,550	17,869	27,766	427
#62	intrasurgical	370	65	260	353	8
#63	operating room	31,707	5,763	56,047	84,900	6,019
#64	real-time	327,719	11,751	797,192	1,302,463	268,351
#65	#59 OR #60 OR #61 OR #62 OR #63 OR #64	638,383	66,511	1,097,625	1,760,092	275,424
#66	#7 AND #58 AND #65	42	0	86	163	0
