# Supplementary material for: Intraoperative Angle Measurement of Anatomical Structures: A Systematic Review
Source: Sensors (Basel). 2024 Mar 1;24(5):1613. doi: 10.3390/s24051613 (PMC10934548; doi:10.3390/s24051613)
Supplement: Supplementary file 1 [file sensors-24-01613-s001.zip › Definitions/logo-mdpi-eps-converted-to.pdf]

MDPI
